# Hygiene for the Aged

**Published:** 1875-11

**Authors:** 


Hygiene for the Aged. — Dr. Habershon speaks of the case of an old man who died simply from the shock produced by going out into the cold and fog, which, though only an inconvenience to people generally, was sufficient to lead to a fatal result in one whose circulation had become enfeebled and whose vital force had so nearly lost its power. The Doctor also alluded to an instance of longevity of which he had been informed by a gentleman—the case being the latter's mother, who had died at the age of 102, and who, during the winter months, used to refuse to get up, saying that she was warm only in bed. To this uniform warm temperature the fact of her great age was doubtless owing, and Dr. Habershon urges that, in prescribing for old people, they should be advised to keep warm; and, as they cannot eat large meals, they should take them more frequently. There are many of them, also, who wake up at about 3 or 4 o'clock in the morning, and it is a good plan for them to have some nourishment then; otherwise the interval between the night and morning meals is too long for their declining strength. The life of the aged may be considerably prolonged by care in these minutiæ.

---

Pay Your Debts.—The ability of nearly every man to pay his debts depends upon his ability to collect debts due him. The individuals of a community are linked together by a chain of debts and credits; and in a time of depression the refusal of a person to discharge a single liability often embarrasses a line of a dozen debtors and creditors. Hence the prompt payment of small debts becomes a public as well as a private duty. The same money that pays a debt in the morning may pay a dozen before night; and twelve men are thus relieved of anxiety and pressure by this payment of one obligation. Pay your debts.

### CRUELTY TO THE INSANE.

An investigation on the part of the State authorities, in regard to the management of the King's County Lunatic Asylum, has been going on for some time, and has revealed instances of shocking brutality by the officers in charge.

A statement was read from Miss Dora B. Robinson, a member of the Local Visiting Committee of the State Aid Association. According to this, a female patient had been strapped into a straight jacket with no clothing but a dress; another female patient had been refused blankets in winter, because she had torn them; delicacies which the former Medical Superintendent gave to the patient, were denied by Dr. Blanchard, the present Medical Superintendent. Mrs. Stribing, a member of the Local Visiting Committee, testified that she had visited the Asylum twice each month, during the past 13 months, to see the German patients. The physicians did not receive her courteously, but sat smoking, with their feet upon a table. A female patient, she said, complained that she had been struck with a stick by a nurse; another said that she had been severely beaten by a nurse. She had seen a very old female patient bleeding from a wound inflicted on her wrist by a nurse, because she refused to be dressed. She had also seen 38 patients in one hall, thinly clad, and suffering from the cold. The food was generally bad, and other supplies were insufficient. There were 204 Germans among the 800 patients, but not enough German nurses, and no German doctors.

Miss Robinson, being recalled, testified that she had seen the steward of the asylum show men through the Asylum, and compel the patients to exhibit themselves like animals; and also that all the nurses were appointed without any regard to their qualifications.

Timothy G. Harley, a former nurse in the Asylum, testified that he had served 24 years as a nurse in different asylums in England and America; he left the King's County Asylum of his own accord in May, 1874, as he was disgusted with its management; he had heard patients complain that their food was bad; Medical Superintendent Blanchard, who took charge of the Asylum in August, 1874, had confessed himself wholly unacquainted with his duties; Dr. Blanchard once neglected to visit his ward for 19 days, and never examined the patients nor inquired about their condition; the attendants were allowed to do as they pleased, and there were no printed rules for their guidance. All the officers, the witness said, from the cook to the Superintendent, kept relatives eating and drinking at the Asylum as long as they liked; he had never seen such a low grade of attendants nor such an undignified and incompetent medical staff; there was no hospital ward for isolating patients suffering from infectious diseases, and a patient sick with typhoid fever had been kept in a ward with 29 other patients, who were physically healthy, in the heat of Summer; there were no night nurses, and sick patients were often found dead in the morning.

What punishment can be meted out to persons guilty of such wickedness, we do not know; but we are certain it will be altogether inadequate to the offences committed against decency and humanity.

